# Postural control and balance in a cohort of healthy people living in Europe: An observational study

**DOI:** 10.1097/MD.0000000000013835

**Published:** 2018-12-28

**Authors:** Antonino Patti, Antonino Bianco, Neşe Şahin, Damir Sekulic, Antonio Paoli, Angelo Iovane, Giuseppe Messina, Pierre Marie Gagey, Antonio Palma

**Affiliations:** a Department of Psychology, Educational Science and Human Movement, University of Palermo, Italy; b Faculty of Sport Science, Ankara University, Turkey; c Faculty of Kinesiology, University of Split, Teslina 6, Split, Croatia; d Department of Biomedical Science, University of Padua, Italy; e Posturalab Italy, Palermo, Italy; f Institut de posturologie, Paris, France; g Regional Sport School of Sicily CONI (Olympic National Italian Committee), Palermo, Italy.

**Keywords:** balance, normative data, normative values, postural control, posturography

## Abstract

In the past 20 years, posturography has been widely used in the medical field. This observational study aimed to report the values derived from posturography of a wide set of healthy subjects from various European countries using a plantar pressure platform and a standardized method of measurement.

A random cluster sampling of 914 healthy subjects aged between 7.0 and 85.99 years, stratified by age, was carried out. To provide percentile values of our cohort, data were processed to obtain 3 curves corresponding to the following percentiles: 25th, 50th, 75th, and the interquartile range. Distance-weighted least squares method was used to represent the percentile on appropriate graphs.

In our sample, the balance to improve with age, up to approximately 45 years, but the trend to reverse with older age. The data show that the oscillations on the sagittal plane (y-mean) change with advancing age. Young people had more retro-podalic support than older people; the balance shifted forward in elderly people.

As the study included a relatively large quantity of data collected using a standardized protocol, these results could be used as normative values of posturography for similar populations. On the basis of this data, correct diagnostic clues will be available to clinicians and professionals in the field. However, further studies are needed to confirm our findings.

## 1 Introduction

The control of standing is a complicated task, and many factors contribute to an adequate postural control. The postural control system is influenced by peripheral sensory systems and their correct functioning.^[1,2]^ The sensory system allows us to perceive the environment and integrate vestibular, visual, and proprioceptive inputs with the central nervous system.^[3,4]^ The literature demonstrated that vision is involved in programming our locomotion,^[5]^ but that subjects with severe sight impairment show an increased somatosensory contribution to balance control.^[6]^ In humans, the sense of balance is governed by postural receptors located in the vestibular, visual, and proprioceptive systems that provide afferent and efferent information to the kinetic muscle chains.^[5,7,8]^ The postural control variables may be reduced when sensory systems are altered.^[9,10]^ A common method of studying standing balance is to record body segment motion equilibrium, which is unstable, and small fluctuations are seen in balance measurements that reflect continuous and intermittent muscle activity.^[11]^ Balance is defined as the maintenance of the vertical projection of the body's center of mass (COM) onto the support area formed by the feet.^[12,13]^ The center of gravity (CoG) is defined as the vertical projection of the COM onto the ground.^[14,15]^ The center of pressure (CoP) is the point of application of the resultant ground reaction force. Winter defined it as the weighted average of all the pressures over the surface of the area in contact with the ground. It is entirely independent of COM.^[5]^ Posturography is aimed at quantifying the body sway of subjects in a standing position.^[16]^ This test records variations of CoP as evidenced on a supporting platform.^[16,17]^ The literature shows that CoP is the primary stabilized reference for posture and movement coordination.^[18]^ CoP can be visualized as 2 shapes: a stabilogram and a statokinesigram. The stabilogram is a representation of CoP displacement in one direction, either anterior–posterior or medial–lateral, presented as a function of time, whereas the statokinesigram is presented in the horizontal plane.^[19,20]^ Over the past 20 years, the posturography has been widely used in many disciplines of medicine .^[21–25]^ In 2016, Kalron et al^[21]^ showed a good correlation between posturography parameters and the Expanded Disability Status Scale parameters. The Expanded Disability Status Scale is an accepted method of quantifying disability in multiple sclerosis and consists of an 8-function system scale monitoring motor, sensory, cerebellar, brain stem, visual, bowel and bladder, pyramidal, and other functions.^[26]^ However, Samson and Crowe^[27]^ established that repeated measurements of the same subjects may show wide ranging values, reflecting high variability. In this context, de Oliveira et al showed that fatigue can interfere with the CoP signal, and this aspect needs to be standardized in the experimental design. On this line, Liu et al^[28]^ described how the feet position can influence the test results, and therefore, all subjects must assume an identical foot position on the platform when evaluated using posturography. In recent years, there has been a surge of interest in low-cost applications to assess balance. Although there is a clear tendency to adopt posturography in daily life to better plan interventions and predict functional disabilities, a major concern at this stage is the high variability associated with the force platform method. Additionally, normative data derived from a large population are missing. Therefore, we assessed postural control and balance in a cohort of healthy people living in Europe to provide normative data derived from posturography, performed with a standardized method. The main aim of this study was to identify values of normality threshold common to all subjects and independent of anthropometric parameters and sex in the sway patterns of healthy subjects during quiet standing.

## 2 Methods

This was an observational study. The study has been retrospectively registered (ISRCTN14957074). The STROBE statement for observational studies was adopted.^[29–31]^ The study design was approved by the Departmental Research Committee (Consiglio di Dipartimento SPPF Prot. n. 290/2014; punto all’ordine del giorno numero 10; approval number: 290–2014/MEDF-02/11), and the subjects were selected according to the criteria approved by the Ethics Committee of the University of Palermo. All the members of the research team were experts in the field (posturologists, physiotherapists, sports science experts). All members coordinated through skype meetings. The sample recruitment was in accordance with both the Italian and Spanish recruitment guidelines. Personal data of participants will be kept confidential. Anthropometric measurements of participants will be provided anonymously.

### 2.1 Anthropometric indices

All measurements were performed twice, and the arithmetic mean was recorded for evaluation. The weight was measured with approximation to 100 g (Wunder 960 classic). Height was measured with a portable Seca stadiometer sensitive to changes up to 1 cm (Seca 220, Hamburg, Germany). Measurements were done with subjects barefoot, the heels, hips, and shoulders touching the stadiometer, and the head in neutral position with eyes gazing forward.^[32,33]^ Data were available after completion of analysis, were stored in the database of our department, and it will be disposed of in 5 years as per university policy. The data were shared anonymously upon request from the researchers with journals and the working research groups. All the data will be linked anonymized. All participants provided informed consent before enrolment. Data were collected from 2014 to 2016. Detailed descriptions of the study sampling and recruitment approaches, standardization, data collection, analysis strategies, quality control activities, and inclusion criteria were approved by all operators who participated in the research. A random cluster sampling of 914 healthy subjects for the observational study, aged between 7.0 and 85 years, stratified by age, was carried out. The inclusion and exclusion criteria were as follows: not having a positive diagnosis for any disease which influences the balance (benign paroxysmal positional vertigo [BPPV], labyrinthitis, Ménière disease, tinnitus, vestibular neuronitis, etc); not ex-professional athletes^[34,35]^; no fracture in the previous 6 months; no falls in the previous 6 months.^[36,37]^ The sample size was calculated with a confidence level of 95% for the ellipse area posturography parameter. Ellipse area/surface quantifies 95% of the total area covered in the medial/lateral and anterior/posterior direction using an ellipse to fit the data.^[17]^ A standardized methodology with standard operating procedures (SOP) has been developed for the data collection^[38–40]^; the standardized methodology was used by all team members. Posturography was performed twice, and scores obtained the second time were used for analysis. For posturography assessment, each participant performed the Romberg test with standardized positioning: feet placed side by side, forming an angle of 30° with both heels separated by 4 cm. Posturography values were measured using the FreeMed posturography system, including the FreeMed baropodometric platform and FreeStep v.1.0.3 software. The sensors, coated with 24 K gold, guaranteed repeatability and reliability of the instrument (Sensor Medica, Guidonia Montecelio, Roma, Italy). After test familiarization, participants were asked to take the standardized Romberg test position on the baropodometric platform. The subjects were barefoot and looking at a specific point with a standardized distance. Data from the platform were converted in accordance with instructions provided by the manufacturer and transformed into coordinates of CoP. The following parameters of the statokinesigram were considered in open eyes conditions: length of sway path of the CoP (SP); ellipse surface area (ES); coordinates of the CoP along the frontal (X; right-left; x-mean), and sagittal (Y; forward-backward; y-mean) planes.^[41]^ The ES and the coordinates along the frontal and sagittal parameters were used and cannot be modified significantly by the sampling rate, according to the 1981 Kyoto conventions.^[16,42]^

### 2.2 Statistical analysis

Analyses were performed using STATISTICA 8.0 for Windows (Statsoft Inc., Tulsa, OK). Statistical significance was set at *P* < .05 for all analyses. We analyzed the normality of variables using the Shapiro–Wilk normality test. Mean and standard deviation (SD) of the measures were calculated, and the difference between sexes was assessed using the Mann–Whitney test. To provide percentile values, sample data were analyzed using maximum penalized likelihood with the LMS statistical method,^[43]^ obtaining 3 curves corresponding to the 25th, 50th, and 75th percentiles, and the interquartile range (IQR). Distance-weighted least squares method was used to represent the percentile on graphs.

### 2.3 Ethics approval and consent to participate

The study design was approved by the Departmental Research Committee (Consiglio di Dipartimento SPPF Prot. n. 290/2014; punto all’ordine del giorno numero 10; approval number: 290–2014/MEDF-02/11), and the subjects were selected according to the criteria approved by the Ethics Committee of the University of Palermo. This study was performed in compliance with the Declaration of Helsinki, the European Union recommendations for Good Clinical Practice (document 111/3976/88, July 1990), and the principles of the Italian data protection act (196/2003) were observed. Informed consent was obtained from all individual participants included in the study.

## 3 Results

**Figure 1:**
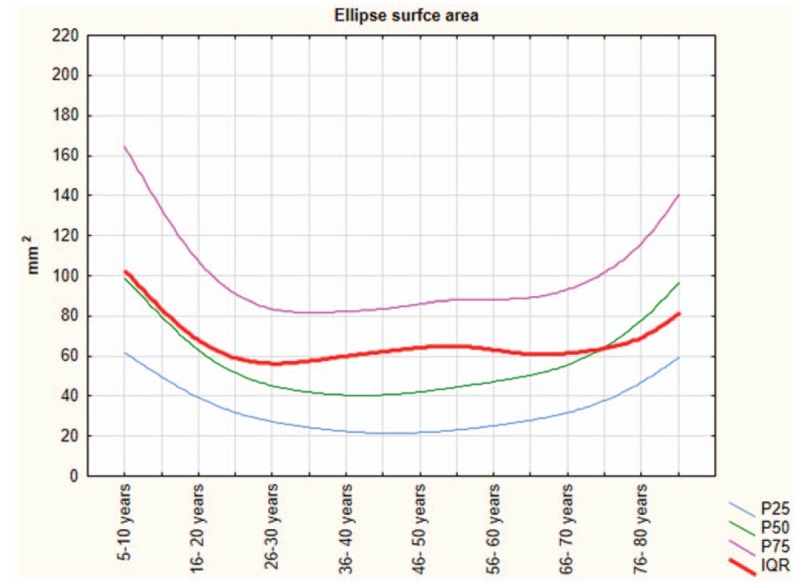
The ellipse surface area parameters.

**Figure 2:**
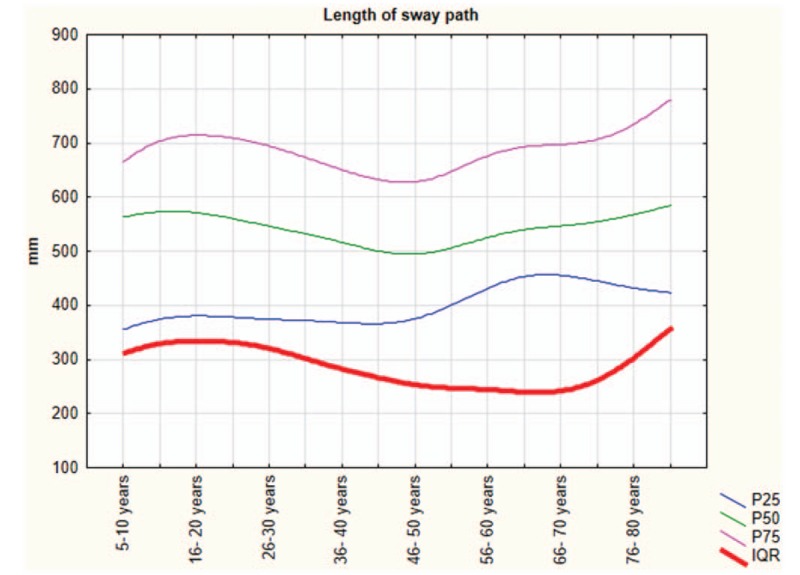
The length of sway path of the coordinates of the center of pressure (CoP).

**Figure 3:**
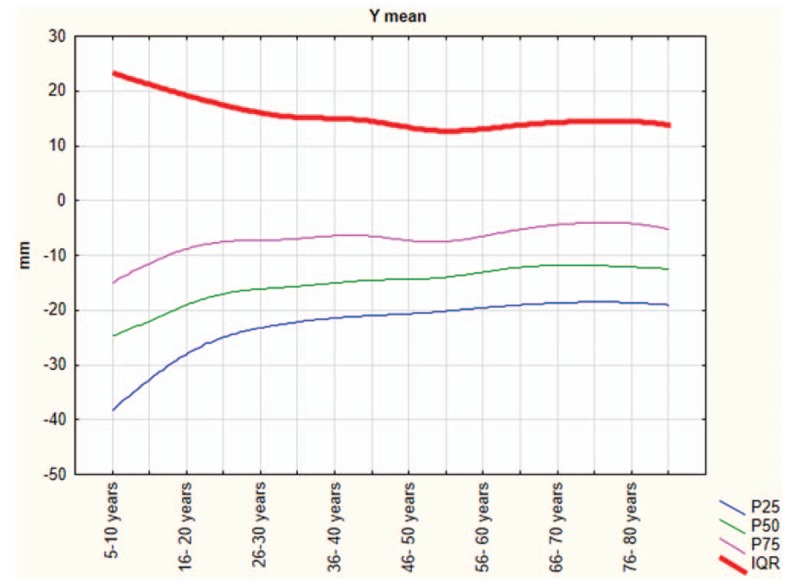
The y-mean parameters.

**Figure 4:**
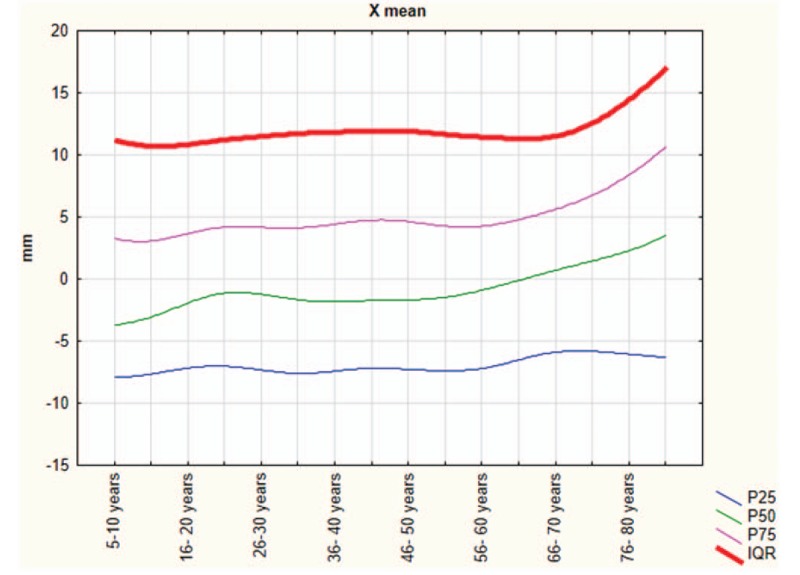
The x-mean parameters.

The Shapiro–Wilk normality test showed that all variables do not assume Gaussian distributions (*P* < .05). Table 1 shows the mean and SD of anthropometric and posturography measures of our sample and statistical analysis to show significant differences using the Mann–Whitney test. Posturography measures did not differ significantly between sexes. Tables 2–5 show the cut-off values of the 25th, 50th, and 75th percentile, and IQR, for each posturography component by age. In our sample, the ES (Table 3 and Fig. 1) improved with age, up to approximately 45 years, but the trend reversed with older age. In addition, the SP analysis was fairly linear, with no clear trend (Table 2 and Fig. 2). Interestingly, the analyses of the y-mean showed an adaptation change with age (Table 5 and Fig. 3). Young people had more retro-podalic support than older people. Although the support remained retro-podalic for all ages, the COP became close to 0 with advancing age. Therefore, the balance shifted forward in elderly people. Similarly, in the analyses of the x-mean, the balance shifted slightly to the right at a young age compared with that at older age (Table 4 and Fig. 4).

**Table 1:**
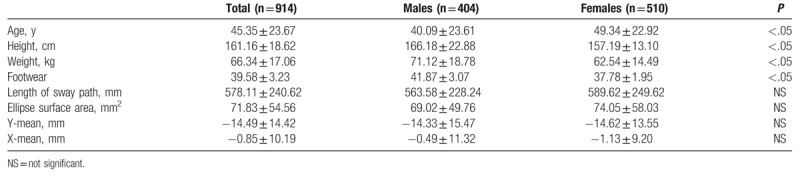
Means and standard deviations of anthropometric and posturography test measures collected in the study sample, by sex.

**Table 2:**
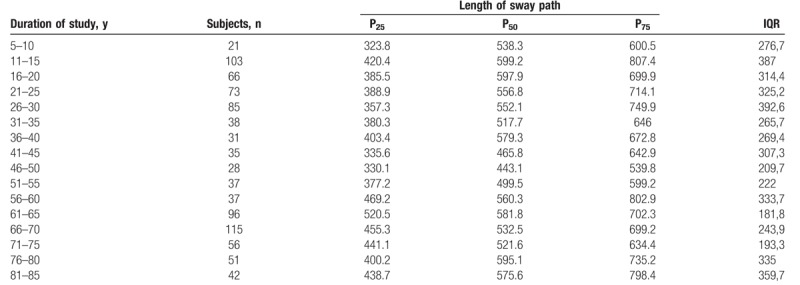
Percentiles and interquartile range (IQR) of the length of sway path (mm).

**Table 3:**
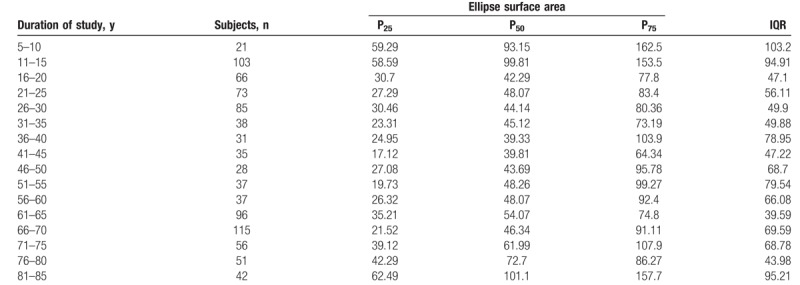
Percentiles and interquartile range (IQR) of the ellipse surface area (mm^2^).

**Table 4:**
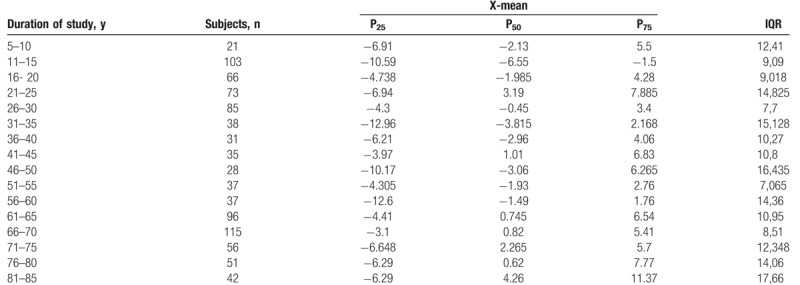
Percentiles and interquartile range (IQR) of the x-mean (mm).

**Table 5:**
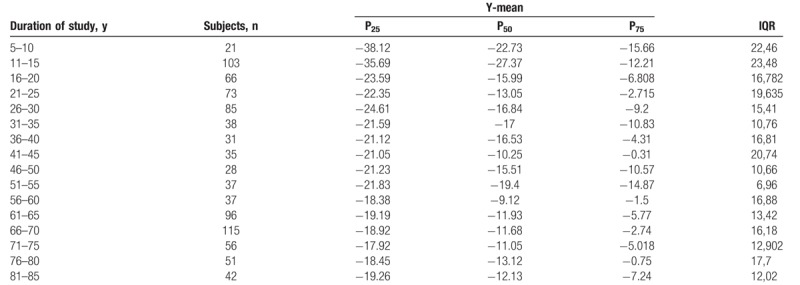
Percentiles and interquartile range (IQR) of the y-mean (mm).

## 4 Discussion

The baropodometric platform is an effective method for measuring postural stability. The main aim of this study was to identify, in healthy subjects and during quiet standing, posturography values of normality threshold, common to all subjects and independent of anthropometric parameters and sex. Some studies enrolled mixed-sex groups that has been shown that COP measures differ between age groups, but the reliability of these measures is not influenced by sexes.^[44,45]^ We have tried to describe in detail the variations of posturography parameters; considering that, especially at a younger or an older age, posturography parameters can vary significantly over 5 years. The 5-year analysis interval was decided after analyzing the literature; the similar study in the literature used the same range.^[46]^ While looking at the optimal age range windows for the observations, a 5-year age range or smaller was already adopted in other investigations.^[47,48]^ To our knowledge, a study with such a large sample and with this type of instrument, on posturography parameters, is the second present in the literature.^[46]^ Similarly, Goble and Baweja^[46]^ reported a relatively high variability of these parameters in the youngest age group (ie, 5–9 years) and in the oldest ones, respectively, but showed a significant improvement on 10 to 14-year-old range and 15 to 19-year-old range, respectively. Subsequently, according to the results of Goble and Baweja,^[46]^ the human balance seems to remain stable until about 50 years then to worsen until the end of life. This conclusion is in line with our findings and confirm our hypothesis. Interestingly, we identified worsening balance in elderly people, and these results are in line with those published in literature.^[49–52]^ However, we also recorded altered balance in young age, probably due to a lack of muscle strength (dynapenia) that is present at this age.^[53]^ Authors reported children's lack of key motor skills (strength, power, coordination) that are necessary components for the balance capacity^[53–56]^; this provides us with a possible explanation of altered balance at early age. The underdeveloped visual-sensory system is another factor that additionally contributes to poorer balance in children.^[57–60]^ Moreover, children have a relatively higher CoG than adults, which consequently alters their balance.^[61]^ These outcomes are in line with the results of Demura et al.^[45]^ The body sway is lower for young adults than preschool children, but is higher for elderly people. Furthermore, in 2014, Barozzi et al showed a similar trend of altered balance in young age, and also, in this case, postural stability improved towards adult age. In younger subjects, a high intersubject variability of stabilometric parameters in comparison to older subjects and adults has been already recorded.^[62,63]^ In a cohort of young 23-year-olds, the study by Clark et al placed the average measure of sway path, measured using the force plate, between the 25th and 75th percentile, as seen in our study, and was very close to the 50th percentile for the corresponding age in our study (410 mm [Clark et al's study] vs 556 mm [in the current study]).^[64]^

Regarding the elderly age, our results indicated a linear decline of balance from ages 70 to 80 years. We retain that the linear decline in cognitive functions and muscle strength/mass seem to be strongly related to postural parameters. Expectedly, these data confirm the main findings in the field of geriatric science.^[65–76]^ In 2017, Blomkvist et al analyzed the reaction time (RT) in a large sample of subjects. The study indicated that the RT gets worse with age.^[77]^ Consequently, the assessment and the modification of the risk are the mainstay of fall prevention in the elderly.^[78]^ In this context, Bianco et al^[79]^ showed that particular physical activity can influence (Dance and ballroom dancing) the RT and, ultimately, decrease the risk falls. In this line, our results could be helpful to prevent these accidental events. On the contrary, in the pediatric age of 5 to 10 years, we again observed the same linear trend probably attributable to the not so consolidated cognition and muscle strength/mass, as mentioned before.^[53,54,80–83]^ The limitations of this study include the use of a single type of stabilometric platform. Although this allowed a homogeneous comparison of all data, further studies with other tools must be carried out to confirm our findings before it can be generalized.

## 5 Conclusions

This study included a relatively large quantity of data collected using a standardized protocol. Therefore, these results could be used as normative values for posturography assessments in similar populations. Because it is evident that the plantar pressure platform method in itself is biased and may interfere with a correct diagnosis of a good or bad posture, we presented percentile values that would be more helpful to professionals in understanding posturography recordings. The study is still ongoing and we aim to recruit a larger population to update these values within the next 5 years.

## Author contributions

Author contributions: Antonino Patti, Pierre Marie Gagey and Antonio Palma designed the study, discussed the results and drafted the paper; Neşe Şahin, Giuseppe Messina performed the testing and participated in drafting paper; Antonio Paoli and Angelo Iovane helped with discussion of results and overviewed previous researches; Damir Sekulic and Antonino Bianco did statistical analyses and drafted the paper.

**Conceptualization:** Antonio Paoli, Antonio Palma.

**Data curation:** Nese Sahin, Angelo Iovane, Giuseppe Messina.

**Formal analysis:** Antonino Bianco, Angelo Iovane.

**Investigation:** Antonino Bianco, Nese Sahin, Damir Sekulic, Antonio Paoli, Angelo Iovane, Giuseppe Messina, Pierre Marie Gagey, Antonio Palma.

**Methodology:** Antonino Patti, Antonino Bianco, Damir Sekulic, Giuseppe Messina, Pierre Marie Gagey, Antonio Palma.

**Supervision:** Nese Sahin, Antonio Paoli, Pierre Marie Gagey, Antonio Palma.

**Validation:** Antonio Palma.

**Visualization:** Damir Sekulic.

**Writing – original draft:** Antonino Patti.

## References

[1] Butler AA Heroux ME Gandevia SC Body ownership and a new proprioceptive role for muscle spindles. Acta Physiol 2017;220:19–27. 10.1111/apha.12792 27561829

[2] Gandevia SC Proprioception, tensegrity, and motor control. J Motor Behav 2014;46:199–201. 10.1080/00222895.2014.883807 24628062

[3] Ruhe A Fejer R Walker B The test-retest reliability of centre of pressure measures in bipedal static task conditions--a systematic review of the literature. Gait Posture 2010;32:436–45. 20947353 10.1016/j.gaitpost.2010.09.012

[4] Proske U Gandevia SC The kinaesthetic senses. J Physiol 2009;587 (Pt 17):4139–46. 19581378 10.1113/jphysiol.2009.175372 PMC2754351

[5] Winter DA Human balance and posture control during standing and walking. Gait Posture 1995;3:193–214.

[6] Kotecha A Webster AR Wright G Standing balance stability and the effects of light touch in adults with profound loss of vision: an exploratory study. Invest Ophthalmol Vis Sci 2016;57:5053–9. 27661857 10.1167/iovs.16-19606

[7] Brandt T Krafczyk S Malsbenden I Postural imbalance with head extension: improvement by training as a model for ataxia therapy. Ann N Y Acad Sci 1981;374:636–49. 6978651 10.1111/j.1749-6632.1981.tb30907.x

[8] Jackson RT Epstein CM Effect of head extension on equilibrium in normal subjects. Ann Otol Rhinol Laryngol 1991;100:63–7. 1985528 10.1177/000348949110000110

[9] Dillon L Clemson L Coxon K Understanding the implementation and efficacy of a home-based strength and balance fall prevention intervention in people aged 50 years or over with vision impairment: a process evaluation protocol. BMC Health Serv Res 2018;18:512. 29970168 10.1186/s12913-018-3304-6 PMC6029014

[10] Dillon CF Gu Q Hoffman HJ Vision, hearing, balance, and sensory impairment in Americans aged 70 years and over: United States, 1999–2006. NCHS Data Brief 2010;1–8. 20377973

[11] Balasubramaniam R Wing AM The dynamics of standing balance. Trends Cogn Sci 2002;6:531–6. 12475714 10.1016/s1364-6613(02)02021-1

[12] Kingma I Toussaint HM Commissaris DA Adaptation of center of mass control under microgravity in a whole-body lifting task. Exp Brain Res 1999;125:35–42. 10100974 10.1007/s002210050655

[13] Barton JE Roy A Sorkin JD An engineering model of human balance control: part I: biomechanical model. J Biomech Eng 2016;138:10.1115/1.4031486 PMC5101042 26328608

[14] Winter DA Prince F Frank JS Unified theory regarding A/P and M/L balance in quiet stance. J Neurophysiol 1996;75:2334–43. 8793746 10.1152/jn.1996.75.6.2334

[15] Jeong H Yamada K Kido M Analysis of difference in center-of-pressure positions between experts and novices during asymmetric lifting. IEEE J Transl Eng Health Med 2016;4:2100311. 27730012 10.1109/JTEHM.2016.2599185 PMC5052028

[16] Kapteyn TS Bles W Njiokiktjien CJ Standardization in platform stabilometry being a part of posturography. Agressologie 1983;24:321–6. 6638321

[17] Paillard T Noe F Techniques and methods for testing the postural function in healthy and pathological subjects. BioMed Res Int 2015;2015:891390. 26640800 10.1155/2015/891390 PMC4659957

[18] Stapley PJ Pozzo T Cheron G Does the coordination between posture and movement during human whole-body reaching ensure center of mass stabilization? Exp Brain Res 1999;129:134–46. 10550511 10.1007/s002210050944

[19] Prieto TE Myklebust JB Hoffmann RG Measures of postural steadiness: differences between healthy young and elderly adults. IEEE Trans Biomed Eng 1996;43:956–66. 9214811 10.1109/10.532130

[20] de Oliveira JM Statokinesigram normalization method. Behav Res Methods 2017;49:310–7. 26896243 10.3758/s13428-016-0706-4

[21] Kalron A Nitzani D Achiron A Static posturography across the EDSS scale in people with multiple sclerosis: a cross sectional study. BMC Neurol 2016;16:70. 27206921 10.1186/s12883-016-0603-6 PMC4873986

[22] Hsu SY Fang TY Yeh SC Three-dimensional, virtual reality vestibular rehabilitation for chronic imbalance problem caused by Meniere's disease: a pilot study. Disabil Rehabil 2016;1–6. 10.1080/09638288.2016.1203027 27418422

[23] Clark RA Seah FJ Chong HC Standing balance post total knee arthroplasty: sensitivity to change analysis from four to twelve weeks in 466 patients. Osteoarthritis Cartilage 2017;25:42–5. 27577930 10.1016/j.joca.2016.08.009

[24] Bremova T Krafczyk S Bardins S Vestibular function in patients with Niemann-Pick type C disease. J Neurol 2016;263:2260–70. 27544496 10.1007/s00415-016-8247-4

[25] Oyarzo CA Villagran CR Silvestre RE Postural control and low back pain in elite athletes comparison of static balance in elite athletes with and without low back pain. J Back Musculoskelet Rehabil 2014;27:141–6. 23963269 10.3233/BMR-130427

[26] Kurtzke JF Rating neurologic impairment in multiple sclerosis: an expanded disability status scale (EDSS). Neurology 1983;33:1444–52. 6685237 10.1212/wnl.33.11.1444

[27] Samson M Crowe A Intra-subject inconsistencies in quantitative assessments of body sway. Gait Posture 1996;4:252–7.

[28] Liu J Sun X Fu M [Influence of foot position in the static posturography]. Lin Chuang Er Bi Yan Hou Ke Za Zhi 2002;16:162–3. 12608280

[29] Moreno-Ramirez D Arias-Santiago S Nagore E [CONSORT, STROBE, and STARD. Tools to improve the reporting of research]. Actas Dermosifiliogr 2015;106:79–81. 25528525 10.1016/j.ad.2014.11.003

[30] Vandenbroucke JP von Elm E Altman DG Strengthening the Reporting of Observational Studies in Epidemiology (STROBE): explanation and elaboration. Int J Surg 2014;12:1500–24. 25046751 10.1016/j.ijsu.2014.07.014

[31] Bolignano D Mattace-Raso F Torino C The quality of reporting in clinical research: the CONSORT and STROBE initiatives. Aging Clin Exp Res 2013;25:9–15. 23740628 10.1007/s40520-013-0007-z

[32] Ozturk A Cicek B Mazicioglu MM Wrist circumference and frame size percentiles in 6-17-year-old turkish children and adolescents in Kayseri. J Clin Res Pediatr Endocrinol 2017;9:329–36. 28515034 10.4274/jcrpe.4265 PMC5785639

[33] Bianco A Mammina C Jemni M A Fitness Index model for Italian adolescents living in Southern Italy: the ASSO project. J Sports Med Phys Fitness 2016;56:1279–88. 26472604

[34] Arkov VV Abramova TF Nikitina TM Comparative study of stabilometric parameters in sportsmen of various disciplines. Bull Exp Biol Med 2009;147:233–5. 19513429 10.1007/s10517-009-0482-6

[35] Paillard T Sport-specific balance develops specific postural skills. Sports Med 2014;44:1019–20. 24668292 10.1007/s40279-014-0174-x PMC4072915

[36] Nonnekes J Goselink RJM Ruzicka E Neurological disorders of gait, balance and posture: a sign-based approach. Nat Rev Neurol 2018;14:183–9. 29377011 10.1038/nrneurol.2017.178

[37] Eibling D Balance disorders in older adults. Clin Geriatr Med 2018;34:175–81. 29661330 10.1016/j.cger.2018.01.002

[38] Patti A Bianco A Paoli A Pain perception and stabilometric parameters in people with chronic low back pain after a pilates exercise program: a randomized controlled trial. Medicine 2016;95:e2414. 26765419 10.1097/MD.0000000000002414 PMC4718245

[39] Patti A Bianco A Messina G The influence of the stomatognathic system on explosive strength: a pilot study. J Phys Ther Sci 2016;28:72–5. 26957731 10.1589/jpts.28.72 PMC4755977

[40] Martines F Messina G Patti A Effects of tinnitus on postural control and stabilization: a pilot study. Acta Med Mediterranea 2015;31:907–12.

[41] Scoppa F Capra R Gallamini M Clinical stabilometry standardization: basic definitions: acquisition interval: sampling frequency. Gait Posture 2013;37:290–2. 22889928 10.1016/j.gaitpost.2012.07.009

[42] Gagey PM Weber B Study of intra-subject random variations of stabilometric parameters. Med Biol Eng Comput 2010;48:833–5. 20582482 10.1007/s11517-010-0656-4

[43] Cole TJ Green PJ Smoothing reference centile curves: the LMS method and penalized likelihood. Stat Med 1992;11:1305–19. 1518992 10.1002/sim.4780111005

[44] Hageman PA Leibowitz JM Blanke D Age and gender effects on postural control measures. Arch Phys Med Rehabil 1995;76:961–5. 7487439 10.1016/s0003-9993(95)80075-1

[45] Demura S Kitabayashi T Noda M Age-stage differences in body sway during a static upright posture based on sway factors and relative accumulation of power frequency. Percept Mot Skills 2008;107:89–98. 18986036 10.2466/pms.107.1.89-98

[46] Goble DJ Baweja HS Normative data for the BTrackS Balance Test of postural sway: results from 16,357 community-dwelling individuals who were 5 to 100 years old. Phys Ther 2018;98:779–85. 29788179 10.1093/ptj/pzy062

[47] Cossio Bolanos M Mendez Cornejo J Luarte Rocha C [Physical activity patterns of school adolescents: validity, reliability and percentiles proposal for their evaluation]. Rev Chil Pediatr 2017;88:73–82. 28288227 10.1016/j.rchipe.2016.07.010

[48] Sarganas G Schaffrath Rosario A Neuhauser HK Resting heart rate percentiles and associated factors in children and adolescents. J Pediatr 2017;187:174–81. e173. 28600156 10.1016/j.jpeds.2017.05.021

[49] Hasselkus BR Shambes GM Aging and postural sway in women. J Gerontol 1975;30:661–7. 1184924 10.1093/geronj/30.6.661

[50] Billot M Simoneau EM Van Hoecke J Age-related relative increases in electromyography activity and torque according to the maximal capacity during upright standing. Eur J Appl Physiol 2010;109:669–80. 20213469 10.1007/s00421-010-1397-7

[51] Melzer I Benjuya N Kaplanski J Postural stability in the elderly: a comparison between fallers and non-fallers. Age Ageing 2004;33:602–7. 15501837 10.1093/ageing/afh218

[52] Goulet ED Melancon MO Lafreniere D Impact of mild hypohydration on muscle endurance, power and strength in healthy, active older men. J Strength Cond Res 2018;32:3405–15. 28234715 10.1519/JSC.0000000000001857

[53] Faigenbaum AD MacDonald JP Dynapenia: it's not just for grown-ups anymore. Acta Paediatr 2017;106:696–7. 28235140 10.1111/apa.13797

[54] Faigenbaum A Resistance exercise and youth: survival of the strongest. Pediatr Exerc Sci 2017;29:14–8. 28271799 10.1123/pes.2016-0262

[55] Faigenbaum AD In response to: keep the physical in physical education. Clin J Sport Med 2017;27:e80. 27755014 10.1097/JSM.0000000000000404

[56] Ratamess NA Kang J Porfido TM Acute resistance exercise performance is negatively impacted by prior aerobic endurance exercise. J Strength Cond Res 2016;30:2667–81. 27658232 10.1519/JSC.0000000000001548

[57] Chia LC Guelfi KJ Licari MK A comparison of the oxygen cost of locomotion in children with and without developmental coordination disorder. Dev Med Child Neurol 2010;52:251–5. 19706141 10.1111/j.1469-8749.2009.03392.x

[58] Wingert JR Welder C Foo P Age-related hip proprioception declines: effects on postural sway and dynamic balance. Arch Phys Med Rehabil 2014;95:253–61. 23994251 10.1016/j.apmr.2013.08.012

[59] Sturnieks DL St George R Lord SR Balance disorders in the elderly. Neurophysiol Clin 2008;38:467–78. 19026966 10.1016/j.neucli.2008.09.001

[60] Dietz V Human neuronal control of automatic functional movements: interaction between central programs and afferent input. Physiol Rev 1992;72:33–69. 1731372 10.1152/physrev.1992.72.1.33

[61] Palmer CE Studies of the center of gravity in the human body. Child Dev 1944;15:99–180.

[62] Baloh RW Fife TD Zwerling L Comparison of static and dynamic posturography in young and older normal people. J Am Geriatr Soc 1994;42:405–12. 8144826 10.1111/j.1532-5415.1994.tb07489.x

[63] Barozzi S Socci M Soi D Reliability of postural control measures in children and young adolescents. Eur Arch Otorhinolaryngol 2014;271:2069–77. 24557440 10.1007/s00405-014-2930-9

[64] Clark RA Bryant AL Pua Y Validity and reliability of the Nintendo Wii Balance Board for assessment of standing balance. Gait Posture 2010;31:307–10. 20005112 10.1016/j.gaitpost.2009.11.012

[65] Kera T Kawai H Yoshida H Physical and psychological characteristics of the community-dwelling elderly with heart disease. Nihon Koshu Eisei Zasshi 2017;64:3–13. 28228632 10.11236/jph.64.1_3

[66] Park KY Hwang HS Kim YP Risk factors for cognitive decline associated with gait speed in community-dwelling elderly Koreans with MMSE scores of 30. Aging Clin Exp Res 2017;29:183–9. 27048507 10.1007/s40520-016-0565-y

[67] Carmel S Raveis VH O’Rourke N Health, coping and subjective well-being: results of a longitudinal study of elderly Israelis. Aging Ment Health 2017;21:616–23. 26829654 10.1080/13607863.2016.1141285

[68] Kang SH Yoon IY Lee SD Subjective memory complaints in an elderly population with poor sleep quality. Aging Ment Health 2017;21:532–6. 26689628 10.1080/13607863.2015.1124839

[69] Lino VT Rodrigues NC O’Dwyer G Handgrip strength and factors associated in poor elderly assisted at a primary care unit in Rio de Janeiro, Brazil. PloS One 2016;11:e0166373. 27832209 10.1371/journal.pone.0166373 PMC5104380

[70] Smolarek Ade C Ferreira LH Mascarenhas LP The effects of strength training on cognitive performance in elderly women. Clin Interv Aging 2016;11:749–54. 27330282 10.2147/CIA.S102126 PMC4896469

[71] Zhou M Peng N Dai Q Effect of Tai Chi on muscle strength of the lower extremities in the elderly. Chin J Integr Med 2016;22:861–6. 26015074 10.1007/s11655-015-2104-7

[72] Morley JE Frailty and sarcopenia in elderly. Wien Klin Wochenschr 2016;128 Suppl 7:439–45. 27670855 10.1007/s00508-016-1087-5

[73] Chen TY Chang HY Developmental patterns of cognitive function and associated factors among the elderly in Taiwan. Sci Rep 2016;6:33486. 27633756 10.1038/srep33486 PMC5025838

[74] Namioka N Sakurai H Terayama H Geriatric problems correlated with cognitive decline using a screening test named “Dr. SUPERMAN” for comprehensive geriatric assessment in elderly inpatients. Geriatr Gerontol Int 2017;17:1252–6. 27489221 10.1111/ggi.12859

[75] Mori K Akezaki Y Role of physical therapists in health care of the elderly. Nihon Eiseigaku Zasshi 2016;71:126–32. 27246151 10.1265/jjh.71.126

[76] Patti A Bianco A Karsten B The effects of physical training without equipment on pain perception and balance in the elderly: a randomized controlled trial. Work 2017;57:23–30. 28506013 10.3233/WOR-172539 PMC5467714

[77] Blomkvist AW Eika F Rahbek MT Reference data on reaction time and aging using the Nintendo Wii Balance Board: a cross-sectional study of 354 subjects from 20 to 99 years of age. PloS One 2017;12:e0189598. 29287063 10.1371/journal.pone.0189598 PMC5747451

[78] Campbell AJ Robertson MC Rethinking individual and community fall prevention strategies: a meta-regression comparing single and multifactorial interventions. Age Ageing 2007;36:656–62. 18056731 10.1093/ageing/afm122

[79] Bianco A Patti A Bellafiore M Group fitness activities for the elderly: an innovative approach to reduce falls and injuries. Aging Clin Exp Res 2014;26:147–52. 24057943 10.1007/s40520-013-0144-4

[80] Moro T Bianco A Faigenbaum AD [Pediatric resistance training: current issues and concerns]. Minerva Pediatr 2014;66:217–27. 24826978

[81] Lloyd RS Cronin JB Faigenbaum AD National Strength and Conditioning Association position statement on long-term athletic development. J Strength Cond Res 2016;30:1491–509. 26933920 10.1519/JSC.0000000000001387

[82] Myer GD Jayanthi N DiFiori JP Sports specialization, part II: alternative solutions to early sport specialization in youth athletes. Sports Health 2016;8:65–73. 26517937 10.1177/1941738115614811 PMC4702158

[83] Faigenbaum AD Lloyd RS MacDonald J Citius, Altius, Fortius: beneficial effects of resistance training for young athletes: narrative review. Br J Sports Med 2016;50:3–7. 26089321 10.1136/bjsports-2015-094621

